# A multi-dimensional phenotyping framework reveals coordinated neuromuscular, immune, and imaging-derived phenotypes in experimental autoimmune myasthenia gravis

**DOI:** 10.3389/fneur.2026.1857888

**Published:** 2026-07-20

**Authors:** Chao Lu, Yongjia Chai, Zhaochi Lyu, Jianyao Wang, Peng Zhang, Peng Zhang

**Affiliations:** 1 Department of Cardiothoracic Surgery, Tianjin Medical University General Hospital, Tianjin, China; 2 Cambridge School of Art, Anglia Ruskin University, Cambridge, United Kingdom

**Keywords:** 18F-FDG PET/CT, EAMG, mouse model, micro-CT, multi-dimensional phenotyping

## Abstract

**Background:**

Experimental autoimmune myasthenia gravis (EAMG) is widely used to investigate the pathogenesis and treatment of myasthenia gravis. However, current evaluation strategies rely predominantly on isolated endpoints and do not fully capture the relationship between neuromuscular dysfunction, immune alterations, and whole-animal phenotypic changes.

**Methods:**

We established EAMG in C57BL/6J mice and implemented a two-tier analytical framework consisting of full-cohort core phenotyping and phenotype-enriched imaging analyses. Core assessments included longitudinal body weight, clinical scoring, electrophysiological testing, behavioral assays, serum anti-AChR levels, and peripheral blood Th17/Treg ratio. In a selectively assessed imaging subset, cardiac ultrasound, micro-CT, and 18F-FDG PET/CT were performed to explore selected imaging-derived cardiac, metabolic, and postural phenotypes. Associations between phenotypic readouts and disease severity were assessed using correlation analyses.

**Results:**

EAMG mice developed consistent neuromuscular and immunological abnormalities, including significant electrophysiological decrement, reduced grip strength and endurance, elevated anti-AChR levels, and increased Th17/Treg ratio. Among these readouts, electrophysiological impairment showed the strongest association with disease severity (rho = −0.79, *P* < 0.001), whereas behavioral and immunological measures demonstrated limited stratification capacity within the EAMG group. In the phenotype-enriched imaging subset, EAMG mice showed reduced cardiac ejection fraction, reduced heart rate, decreased iliopsoas FDG uptake, and reduced sagittal spinal angle. Iliopsoas FDG uptake was negatively correlated with disease severity within this subset (rho = −0.89, *P* = 0.041).

**Conclusions:**

EAMG was associated with coordinated electrophysiological, functional, and immunological abnormalities in the full cohort. Exploratory imaging analyses in a phenotype-enriched subset further identified selected cardiac, metabolic, and postural phenotypes in animals with overt disease manifestations. This multi-dimensional framework may support more comprehensive assessment of disease progression in preclinical myasthenia gravis studies, while the imaging-derived findings require validation in unselected cohorts.

## 1 Introduction

Myasthenia gravis (MG) is an antibody-mediated autoimmune disorder characterized by impaired neuromuscular transmission caused by pathogenic autoantibodies directed against acetylcholine receptors (AChR) or related molecules at the postsynaptic membrane of the neuromuscular junction (1–3). According to the underlying autoantibody profile, MG can be broadly classified into AChR-positive, MuSK-positive, LRP4-positive, and seronegative subtypes, each of which differs in immunopathogenesis and clinical behavior (2, 4–6). Although current therapies, including thymectomy, corticosteroids, immunosuppressants, and targeted biologics, have improved outcomes, MG remains a heterogeneous disease in both severity and therapeutic response (2, 4). Experimental autoimmune myasthenia gravis (EAMG) remains a widely used and well-established model for mechanistic and translational studies of MG. Since the original induction of EAMG by immunization with purified AChR, the model has played a central role in investigations of autoimmune mechanisms, neuromuscular junction pathology, and therapeutic intervention (7, 8). EAMG reproduces key features of AChR antibody-positive MG, including the generation of pathogenic autoantibodies, electrophysiological abnormalities, and clinical manifestations of muscle weakness (7, 9, 10). As a result, it has become one of the most widely used *in vivo* platforms for mechanistic and translational studies in MG. Despite its extensive use, evaluation of EAMG still relies largely on a relatively limited set of endpoints. Clinical scoring systems such as the Lennon score are practical but inevitably influenced by observer judgment and by the behavioral variability inherent to small animals (11, 12). Electrophysiological and immunological assays provide important mechanistic information, yet they often capture only one aspect of disease burden and do not fully reflect whole-animal functional status. Consequently, current EAMG assessment strategies are useful for confirming model establishment but are less suited to capturing disease-related changes at the whole-animal level. In parallel, imaging-based phenotyping has become increasingly important in both clinical and preclinical research. Ultrasound can provide non-invasive assessment of cardiac structure and function, CT enables objective evaluation of anatomical alignment and structural change, and PET/CT offers quantitative information on tissue metabolism (13–20). These modalities are particularly attractive in animal studies because they provide integrative, organ-level readouts that are difficult to obtain from conventional clinical scoring alone. In principle, such approaches could complement classical EAMG endpoints by capturing additional whole-animal phenotypic readouts that are not available from conventional scoring, electrophysiology, or serological assays alone. However, whether EAMG can be assessed through a multi-dimensional phenotyping framework that integrates neuromuscular, immune, and imaging-derived readouts remains insufficiently explored (19). It is not yet clear whether electrophysiological impairment, behavioral dysfunction, immune activation, and selected imaging-derived abnormalities are coordinated within the same disease context. Addressing this question is important, because a more integrated phenotypic framework could improve both mechanistic interpretation and the evaluation of therapeutic interventions in EAMG. In the present study, we therefore applied a two-tier analytical strategy combining full-cohort core phenotyping with phenotype-enriched imaging analyses. By integrating electrophysiological, functional, immunological, and imaging-derived readouts, we aimed to determine whether core EAMG phenotypes show coordinated changes and whether selected exploratory imaging-derived phenotypes are associated with disease severity.

## 2 Materials and methods

### 2.1 Study design and analytical framework

This study was an animal experiment based on the classical experimental autoimmune myasthenia gravis (EAMG) model and was conducted using a two-tier analytical framework consisting of full-cohort core phenotyping and phenotype-enriched subset-based extended phenotyping. The first tier comprised full-cohort analyses. All mice included in the modeling process were used for post-induction assessment of core EAMG phenotypes, including body weight change, clinical scoring, behavioral testing, electrophysiological evaluation, and serum anti-AChR antibody measurement. These analyses were intended to determine whether the model had been successfully established and to define its canonical neuromuscular junction-related phenotype. The second tier comprised exploratory extended phenotypic analyses. Because some imaging assessments were costly and experimental resources were limited, echocardiography and PET/CT were not performed in all animals. Instead, after completion of the core phenotypic assessment, a selectively assessed imaging subset was established by preferentially including EAMG mice with overt disease manifestations, together with contemporaneous control animals. This subset was used to explore selected imaging-derived phenotypes related to cardiac function, skeletal muscle metabolism, and sagittal spinal posture. Analyses of this imaging subset were intended to generate hypotheses regarding extended phenotypes and were not used to replace the primary conclusions drawn from the full cohort. The prespecified objective was to assess the concordance of electrophysiological, functional, and immunological abnormalities in the full EAMG cohort. A secondary exploratory objective was to determine whether, in a phenotype-enriched imaging subset, disease severity was accompanied by selected imaging-derived cardiac, metabolic, and postural phenotypes. The primary endpoints were electromyographic decrement, grip strength, hanging endurance, and serum anti-AChR antibody level at day 90. Exploratory extended endpoints included echocardiographic parameters, PET/CT-derived metabolic indices, and sagittal spinal angle.

### 2.2 Sample size considerations

Sample size considerations were defined separately for the full-cohort core phenotyping and the exploratory imaging subset. The full cohort included 15 mice per group, which is consistent with sample sizes commonly used in previous EAMG studies with comparable immunization-based experimental designs (11, 12). No formal *a priori* power calculation was performed for the full cohort, because this study was designed as an integrative phenotyping investigation rather than a confirmatory interventional trial based on a single primary efficacy endpoint. The sample size was therefore determined by prior experimental practice in EAMG research, feasibility of completing multi-domain phenotyping, and animal welfare considerations. The imaging subset size (5 EAMG mice and 6 CFA mice) was determined by the high cost and logistical constraints of echocardiography, PET/CT, and micro-CT acquisition, combined with the phenotype-enriched imaging strategy described below. This subset was not designed or powered for definitive group-level inference. Accordingly, all imaging-derived analyses were interpreted as exploratory and hypothesis-generating throughout the manuscript.

### 2.3 Experimental animals

Female C57BL/6J mice aged 6–8 weeks and weighing 22–24 g were purchased from Huafukang Bioscience Co., Ltd. (Beijing, China). All animals were housed under specific pathogen-free conditions in an independent ventilated cage system, with a 12-h light/dark cycle, ambient temperature maintained at 23 ± 2 °C, and free access to food and water. Once overt disease manifestations appeared, softened food and hydrogel were continuously provided on the cage floor to minimize the impact of feeding difficulty on general condition. All animal procedures were performed in accordance with relevant Chinese regulations and institutional standards for animal welfare and ethics, with efforts made to minimize animal use and unnecessary suffering.

### 2.4 EAMG induction and experimental grouping

Mice were randomly assigned to two groups: an EAMG group and a complete Freund's adjuvant (CFA) control group, with 15 animals in each group. The EAMG model was induced by immunization with the AChR R97–116 peptide. Under 2% isoflurane inhalation anesthesia, mice received subcutaneous injections of an emulsion containing 20 μg of AChR R97–116 peptide in CFA, with a total injection volume of 200 μL, distributed across the footpad and six separated sites on the back. Booster immunizations were administered on days 30 and 60 at the tail base and three separated sites on the back using the same dose of peptide emulsified in incomplete Freund's adjuvant (IFA). CFA control mice underwent the same immunization schedule and injection volume but received Freund's adjuvant emulsified with PBS alone, without the R97–116 peptide.

### 2.5 Clinical observation, body weight monitoring, and disease severity stratification

All animals were monitored continuously from the time of first immunization. Body weight was recorded every 2 days. Clinical severity was evaluated weekly in a blinded manner using the Lennon scoring system:

- 0, Normal muscle strength, with no weakness even after repeated gripping exercise.
- 1, normal at rest but weakness after exercise, manifested as chin-on-floor posture, difficulty raising the head, hunching, and reduced mobility.
- 2, overt weakness at rest.
- 3, moribund state with dehydration or quadriplegia.
- 4, death.

In the full-cohort analyses, Lennon scores were used to confirm model establishment and to describe disease evolution. Within the EAMG group, end-point scores were further used for severity stratification to assess the relationship between disease severity and multidimensional phenotypic measures. Control animals were not included in severity-stratified analyses.

### 2.6 Grip strength and hanging endurance tests

To assess disease-related functional impairment, grip strength and hanging endurance were evaluated at the terminal time point under the same experimental conditions for all animals. Hanging endurance was quantified using an inverted grid test. Each mouse was placed at the center of a metal grid (50 cm in length; mesh spacing, 1 cm). After the animal had grasped the grid, the grid was rapidly inverted and maintained in a horizontal position above cushioning material to prevent injury from falling. The latency to fall was recorded in seconds as the hanging endurance time. Mice that remained on the grid for the full observation period were assigned the maximum value of 600 s. Grip strength was measured using a YLS-13A force meter (Jinan Yiyan Technology Development Co., Ltd., China). Before testing, mice underwent a mild exercise challenge similar to that used during clinical scoring to increase sensitivity to disease-related weakness. Each mouse was allowed to grasp the force meter grid, and the animal was then gently pulled backward until it released its grip. The peak force displayed by the instrument was recorded in grams as the grip strength value. For both behavioral assays, the individual mouse was used as the statistical unit, and the terminal day 90 value was used for subsequent analysis.

### 2.7 Repetitive nerve stimulation electromyography

At the terminal time point, repetitive nerve stimulation electromyography was performed. Mice were anesthetized by intraperitoneal injection of sodium pentobarbital (65 mg/kg). A ground electrode was placed on the anterior chest skin; the stimulating positive electrode was positioned near the sciatic notch to stimulate the sciatic nerve, and the negative electrode was placed subcutaneously in the abdominal wall. The recording electrode was inserted into the ipsilateral gastrocnemius muscle, with the reference electrode placed at the gastrocnemius tendon. Ten supramaximal stimuli were delivered at frequencies of 3, 5, and 10 Hz, with a pulse duration of 0.2 ms, and compound muscle action potential changes were recorded. Neuromuscular transmission was assessed based on the decrement of waveform amplitude across the stimulation train. A representative decremental parameter at the terminal time point was used for statistical analysis.

### 2.8 Serum anti-AChR antibody assay

At the terminal time point, peripheral blood was collected and serum was isolated for measurement of anti-AChR R97–116 IgG by enzyme-linked immunosorbent assay (ELISA). Ninety-six-well plates were coated overnight at 4 °C with 100 μL of R97–116 peptide at 5 μg/ml. Plates were then blocked for 2 h at 37 °C with PBS containing 0.05% Tween 20 and 10% fetal bovine serum. Serum diluted 1:100 was added at 100 μL per well and incubated for 2 h at 37 °C. After washing, HRP-conjugated rabbit anti-mouse IgG diluted 1:2000 was added and incubated at room temperature for 1 h. TMB substrate was then applied, and absorbance was measured at 450 nm. Results were expressed as optical density (OD) values.

### 2.9 Flow cytometry analysis of peripheral blood Th17 and Treg cells

Peripheral blood Th17 and Treg cell populations were analyzed by flow cytometry at the terminal time point. For Th17 analysis, 250 μL of EDTA-anticoagulated peripheral blood was mixed with an equal volume of RPMI-1640 medium and stimulated with 1 μL of an anti-CD3/CD28-based lymphocyte stimulation reagent at 37 °C in 5% CO_2_ for 4 h. After stimulation, 250 μL of the cell suspension was transferred to a 1.5-mL tube and stained with FITC anti-mouse CD4 antibody. For Treg analysis, 250 μL of EDTA-anticoagulated peripheral blood was directly stained with FITC anti-mouse CD4 and APC anti-mouse CD25 antibodies without *ex vivo* stimulation. Surface staining was performed at room temperature in the dark for 20 min. After surface staining, red blood cells were lysed with 1 ml of 1 × red blood cell lysis buffer for 10 min at room temperature in the dark, followed by centrifugation at 2,500 rpm for 5 min. Cells were then fixed and permeabilized using Foxp3 permeabilization reagent for 30 min at room temperature in the dark. After washing with 1 × buffer, intracellular staining was performed using PerCP/Cyanine5.5 anti-mouse IL-17A antibody for Th17 detection or PE anti-mouse Foxp3 antibody for Treg detection, with incubation at room temperature in the dark for 1 h. After washing with PBS, cells were resuspended in 300 μL PBS and analyzed using a BD flow cytometer. Lymphocytes were first gated according to forward scatter and side scatter characteristics. Th17 cells were defined as CD4^+^IL-17A^+^ cells within the lymphocyte gate. Treg cells were defined as CD4^+^CD25^+^Foxp3^+^ cells. The percentages of Th17 and Treg cells were calculated for each animal, and the Th17/Treg ratio was used as an index of peripheral T-cell immune balance. The antibody panel included FITC anti-mouse CD4 (clone GK1.5, BioLegend, Cat#100405), APC anti-mouse CD25 (clone 3C7, BioLegend, Cat#101909), PerCP/Cyanine5.5 anti-mouse IL-17A (clone TC11-18H10.1, BioLegend, Cat#506919), and PE anti-mouse Foxp3 (clone MF-14, BioLegend, Cat#126403).

### 2.10 Micro-CT and measurement of sagittal spinal angle

To evaluate disease-associated postural abnormalities, micro-CT was performed in a subset of animals. Under isoflurane inhalation anesthesia (2% for induction and 1.2%−1.5% for maintenance), mice were placed in the prone position in an InLiview3000B animal CT system (Beijing Yongxin Medical Equipment Co., Ltd., China). Scan settings included a 0.5-mm aluminum filter, 80 kV, and 0.5 mA. Axial images were reconstructed using NMSoft-AIWS software, and sagittal reconstructions were generated using CT-VR software. Sagittal spinal alignment angles were then measured using ImageJ (NIH, version 1.53). Because this parameter reflects imaging-based quantification of disease-associated posture rather than true osseous deformity, it was defined in this study as the sagittal spinal angle, rather than interpreted as structural skeletal malformation.

### 2.11 Echocardiography

Cardiac ultrasound examinations were performed using a Philips EPIQ 7 system (Philips Ultrasound, Amsterdam, The Netherlands) equipped with a 5–12 MHz phased-array transducer. Mice were maintained under continuous 1.5% isoflurane anesthesia in the supine position. After chest hair removal, the transducer was placed at the standard left parasternal position to obtain cardiac images. The following parameters were recorded: ejection fraction (EF), fractional shortening (FS), left ventricular internal diameter at end systole (LVIDs), left ventricular internal diameter at end diastole (LVIDd), end-diastolic volume (EDV), end-systolic volume (ESV), cardiac output (CO), and heart rate (HR). Because of the expense and logistical demands of this examination, and because the aim was to explore whether animals with evident disease manifestations showed additional imaging-derived cardiac phenotypes, echocardiography was not performed in all animals. Instead, it was conducted within the phenotype-enriched subset after completion of core phenotypic assessment.

### 2.12 18F-FDG PET/CT

PET/CT imaging was performed using an InLiview3000B animal PET/SPECT/CT system (Beijing Yongxin Medical Equipment Co., Ltd., China). Mice were fasted for 12 h before imaging, with free access to water. Under isoflurane anesthesia (2% induction, 1.5% maintenance), 100 μCi of 18F-FDG in approximately 100 μL PBS was administered via tail vein injection. PET acquisition began 50 min after tracer injection. CT scanning was performed for anatomical localization and attenuation correction using the following parameters: 80 kV, 0.5 mA, 0.8-mm slice thickness, 40 iterations, and spiral acquisition mode. Body temperature was maintained at approximately 37 °C throughout the procedure. Based on reconstructed images, predefined regions of interest (ROIs) were used to quantify metabolic parameters in skeletal muscle and myocardium, with particular attention to paraspinal and iliopsoas regions. As with echocardiography, PET/CT was performed in the phenotype-enriched subset to explore selected skeletal muscle and myocardial metabolic phenotypes in EAMG.

### 2.13 Composition and interpretation of the imaging subset

Because echocardiography and PET/CT were high-cost imaging procedures and could not be performed in all animals, a phenotype-enriched imaging strategy was used. Specifically, after completion of core phenotypic assessment, EAMG mice with clear disease manifestations were preferentially selected for imaging evaluation, including those with increased clinical scores, reduced grip strength, shortened hanging endurance, or electrophysiological abnormalities. Contemporaneous CFA animals were included as controls. The final imaging subset comprised 5 EAMG mice and 6 CFA mice. This subset was non-random and enriched for animals with more evident disease phenotypes. The purpose of this subset analysis was to identify selected imaging-derived phenotypes involving cardiac function, skeletal muscle metabolism, and sagittal posture, rather than to estimate unbiased cohort-wide effect sizes or determine the prevalence of organ-level involvement in all EAMG animals. Accordingly, all imaging findings were interpreted as exploratory and hypothesis-generating and were considered only in conjunction with the full-cohort neuromuscular and immunological results.

### 2.14 Data integration and statistical analysis

#### 2.14.1 Full-cohort analysis

Full-cohort analysis included all animals with available core phenotypic data. This layer of analysis was used to compare EAMG and CFA groups with respect to classical disease endpoints, including body weight change, electrophysiological variables, behavioral measures, and serum antibody levels. Because body weight was repeatedly measured over time, a longitudinal analytical strategy was used to evaluate the effects of group, time, and their interaction on body weight trajectories. Because body weight was repeatedly measured over time, a longitudinal analytical strategy was used to evaluate the effects of group, time, and their interaction on body weight trajectories. Other terminal continuous variables were compared between groups using parametric or nonparametric methods as appropriate to the distribution of the data.

#### 2.14.2 Severity analysis within the EAMG group

To assess the relationship between disease severity and multidimensional phenotypic measures, severity analyses were performed within the EAMG group based on the Lennon score. Because the score is ordinal in nature and subgroup sizes were unbalanced, rank-based correlation analysis was prioritized over simple comparisons of group means.

#### 2.14.3 Imaging subset analysis

Imaging subset analyses were restricted to animals that underwent echocardiography, PET/CT, or micro-CT. Given the small sample size and non-random composition of this subset, all related analyses were considered exploratory. Continuous variables were preferentially compared between EAMG and CFA groups using nonparametric methods, and associations between imaging parameters and Lennon score were further evaluated within the EAMG subset.

#### 2.14.4 Missing data and statistical unit

Not all modalities were completed in all animals; therefore, actual sample sizes varied across variables. All analyses were conducted using available-case data only, and no imputation of missing values was performed. The statistical unit was defined uniformly as the individual animal. Multiple imaging regions or repeated measurements from the same animal were not treated as independent observations. For variables lacking confirmed one-to-one cross-modal matching, mouse-level cross-modal correlation analyses were not performed to avoid pseudoreplication and spurious inference.

#### 2.14.5 Statistical software and significance threshold

All statistical analyses were performed in R software (R version 4.5.1). Continuous variables are presented as mean ± standard deviation or median (interquartile range), as appropriate. All tests were two-sided, and a *P* value < 0.05 was considered statistically significant. Because imaging subset analyses were exploratory in nature, greater emphasis was placed on effect direction, distributional pattern, and consistency with disease severity than on nominal significance thresholds alone.

## 3 Results

**Figure 1:**
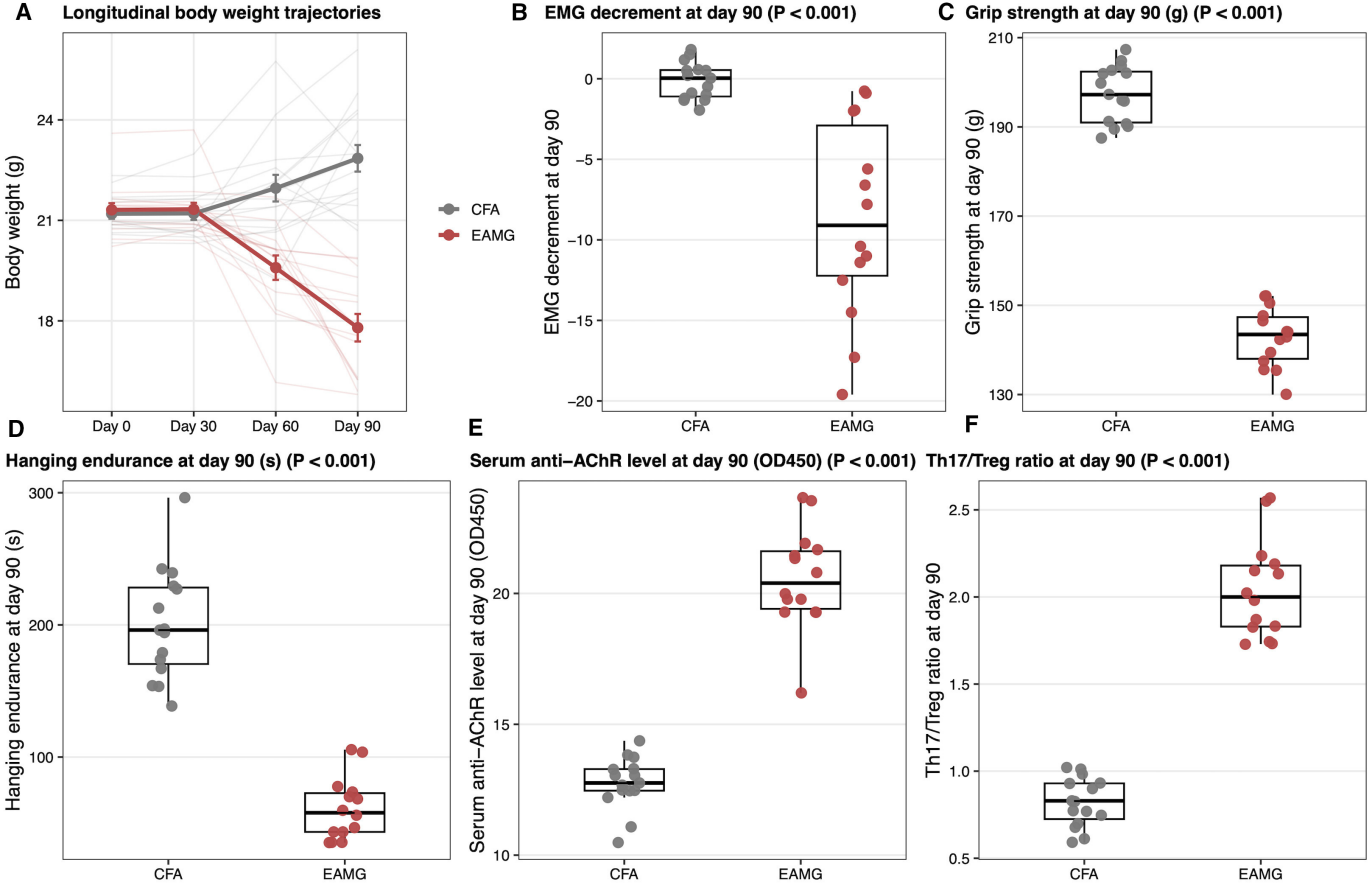
Establishment of the EAMG model and core phenotypic characterization. Longitudinal and terminal assessments of EAMG and CFA control mice. **(A)** Body weight trajectories over time, showing divergence between groups after day 30. **(B)** Electromyographic decrement at day 90. **(C)** Grip strength measured at the terminal time point. **(D)** Hanging endurance assessed by inverted grid test. **(E)** Serum anti-AChR antibody levels measured by ELISA. **(F)** Peripheral blood Th17/Treg ratio determined by flow cytometry. Th17 cells were defined as CD4^+^IL-17A^+^ cells, and Treg cells were defined as CD4^+^CD25^+^Foxp3^+^ cells. Data are presented as mean ± SD or median (IQR) as appropriate. Each dot represents one animal. Statistical comparisons between groups were performed using parametric or nonparametric tests as appropriate.

Establishment of the EAMG model and core phenotypic characterization: Longitudinal analysis showed that body weight trajectories were comparable between groups at baseline and early time points, but diverged progressively after day 30, with a marked decrease in the EAMG group by day 90 (Figure 1A). Mixed-effects modeling confirmed a significant group-by-time interaction, indicating distinct disease trajectories. At the terminal time point, the results were directionally consistent across multiple readouts. Compared with CFA controls, EAMG mice exhibited a significantly greater decremental response on electromyography, markedly reduced grip strength, and substantially shortened hanging endurance (Figures 1B–D), together indicating impaired neuromuscular transmission at both electrophysiological and functional levels. In parallel, EAMG mice showed significantly elevated serum anti-AChR levels and an increased peripheral blood Th17/Treg ratio (Figures 1E, F), suggesting concomitant humoral immune activation and T-cell immune imbalance. Overall, electrophysiological, behavioral, and immunological readouts changed in the same direction, indicating that the model captured a coordinated disease phenotype rather than isolated endpoint-specific abnormalities. Representative images and traces illustrating selected EAMG features, including posture abnormalities, decremental electromyographic responses, sagittal spinal alignment, and FDG uptake patterns, are shown in Supplementary Figure S1.

**Figure 2:**
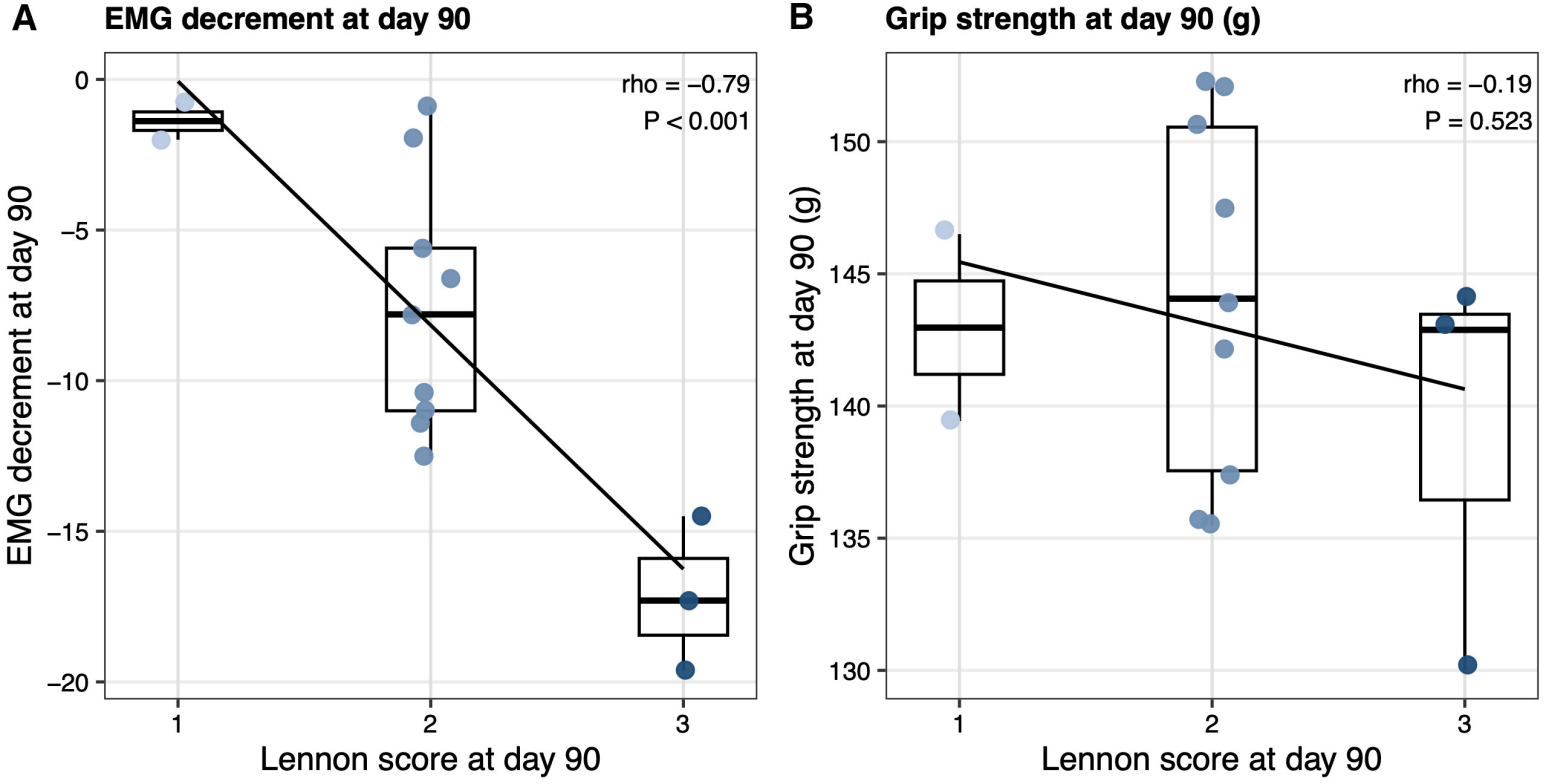
Association between disease severity and core phenotypic readouts in EAMG mice. Correlation analyses between Lennon score at day 90 and functional parameters within the EAMG group. **(A)** Correlation between Lennon score and electromyographic decrement. **(B)** Correlation between Lennon score and grip strength. Each dot represents one animal. Correlation coefficients were calculated using Spearman's rank correlation. Exact rho and *P* values are indicated in the plots.

Association between disease severity and core phenotypic readouts: Within the EAMG group, disease severity was most strongly associated with electrophysiological impairment. At day 90, Lennon score showed a significant negative correlation with electromyographic decrement (rho = −0.79, *P* < 0.001) (Figure 2A). In contrast, grip strength was only weakly correlated with disease severity and did not reach statistical significance (rho = −0.19, *P* = 0.523) (Figure 2B). Similarly, hanging endurance showed no significant association (rho = 0.19, *P* = 0.524) (Supplementary Figure S2A). In addition, neither the Th17/Treg ratio nor serum anti-AChR level was significantly associated with disease severity (Supplementary Figures S2B–C). These findings suggest that behavioral and selected immunological measures discriminate well between groups but are less informative for severity stratification within the EAMG group. Accordingly, electromyographic decrement was used as the primary reference measure for severity stratification, while behavioral and immunological variables were interpreted as complementary phenotypes. Full correlation statistics are summarized in Supplementary Table S2.

**Figure 3:**
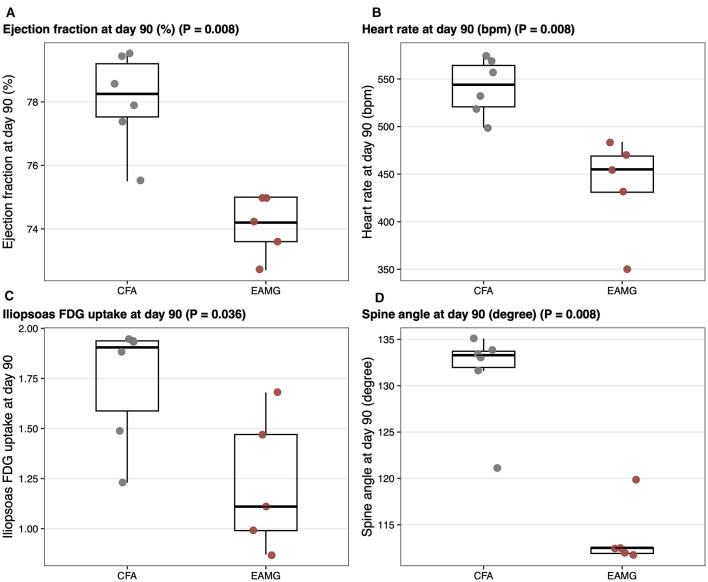
Exploratory imaging-derived phenotypes in the phenotype-enriched subset. Selected imaging-derived cardiac, metabolic, and postural readouts in EAMG and CFA mice assessed in a phenotype-enriched subset. **(A)** Left ventricular ejection fraction measured by echocardiography. **(B)** Heart rate measured during echocardiographic acquisition. **(C)** Iliopsoas muscle FDG uptake quantified by PET/CT. **(D)** Sagittal spinal angle measured by micro-CT. Each dot represents one animal. Continuous variables were compared using nonparametric tests. These analyses were exploratory and were not intended to estimate cohort-wide effect sizes.

Exploratory imaging-derived phenotypes in the phenotype-enriched subset: In the phenotype-enriched imaging subset, EAMG mice showed directionally consistent differences in selected imaging-derived readouts. Compared with CFA controls, EAMG mice showed reduced ejection fraction and reduced heart rate (Figures 3A, B), decreased iliopsoas FDG uptake (Figure 3C), and reduced sagittal spinal angle (Figure 3D). These observations suggest that, among animals selected for imaging, overt EAMG phenotypes may be accompanied by additional cardiac, metabolic, and postural changes. Because imaging was performed in a selectively assessed subset rather than the full cohort, these findings should be regarded as exploratory and should not be interpreted as cohort-wide estimates of organ-level involvement. Within this subset, correlation analyses revealed that iliopsoas FDG uptake was negatively associated with disease severity (rho = −0.89, *P* = 0.041), whereas no significant associations were observed for cardiac or structural parameters (Supplementary Figures S3A–D). These findings are summarized in Supplementary Table S3.

## 4 Discussion

In this study, we applied an integrated phenotyping strategy to evaluate EAMG using classical neuromuscular, behavioral, immunological, and selected imaging-derived readouts. Full-cohort analyses confirmed successful model establishment and showed that electromyographic decrement was the readout most closely associated with disease severity. In a phenotype-enriched imaging subset, additional differences were observed in cardiac function, skeletal muscle metabolism, and sagittal posture. Because this subset was non-randomly selected for animals with more evident disease manifestations, the imaging-derived findings should be interpreted as exploratory and require validation in unselected cohorts. The classical features of EAMG observed in our study are consistent with the established biology of the model. Immunization with AChR peptide induced overt clinical weakness, electrophysiological decrement, and a marked increase in serum anti-AChR levels, indicating successful induction of an autoimmune response directed against the neuromuscular junction (9, 21, 22). The increase in the Th17/Treg ratio further supports the presence of immune dysregulation accompanying disease induction, in line with prior studies showing that T-cell subset imbalance contributes to EAMG pathogenesis and may be therapeutically modifiable (23–25). Importantly, these electrophysiological, functional, and immunological changes were directionally aligned rather than isolated, suggesting that the model captured a coherent disease state rather than fragmented readouts from unrelated assays. A central observation in the present work is that disease severity, as defined by the Lennon score, was most closely linked to electrophysiological impairment. The strong negative correlation between score and electromyographic decrement indicates that repetitive nerve stimulation remains one of the most stable functional indices of disease progression within EAMG. In contrast, grip strength and hanging endurance, although highly effective for distinguishing EAMG mice from controls, showed limited stratification capacity within the EAMG group itself. This difference is biologically plausible. Electrophysiological readouts more directly reflect neuromuscular junction transmission failure, whereas behavioral performance is influenced by a broader set of variables, including fatigue, motivation, postural compensation, and whole-animal condition. From a practical standpoint, these findings suggest that behavioral assays are valuable for model-level discrimination, but electrophysiological measures may provide a more reliable axis for severity stratification. An additional exploratory aspect of this study was the application of clinically relevant imaging modalities to extend EAMG phenotyping beyond conventional core endpoints. In the phenotype-enriched subset, EAMG mice showed reduced ejection fraction, reduced heart rate, reduced iliopsoas FDG uptake, and decreased sagittal spinal angle compared with CFA controls. Although these endpoints span different physiological domains, their directionality was internally consistent. This pattern raises the possibility that animals with more evident EAMG phenotypes may show additional physiological changes that are not captured by clinical scoring and electrophysiology alone. Among the imaging-derived measures, reduced skeletal muscle metabolic activity may be the most intuitively linked to EAMG pathophysiology. Impaired neuromuscular transmission could plausibly reduce effective muscle activation and contractile demand, thereby contributing to lower glucose utilization in affected muscle groups. The inverse correlation between iliopsoas FDG uptake and Lennon score is consistent with a potential link between neuromuscular impairment and skeletal muscle metabolism in this phenotype-enriched subset. Nevertheless, this association should be viewed cautiously. Because this analysis was based on a small, selectively assessed subset, it should be considered hypothesis-generating rather than definitive. The cardiac findings require particularly cautious interpretation. EAMG mice in the phenotype-enriched imaging subset exhibited lower ejection fraction and lower heart rate relative to CFA controls. However, the present study does not establish primary myocardial involvement or a direct autoimmune cardiac mechanism. These differences may reflect secondary physiological effects related to disease severity, reduced activity, altered autonomic state, anesthesia, or general systemic condition. We therefore interpret these cardiac measurements as exploratory physiological readouts rather than evidence of direct cardiac involvement. Dedicated mechanistic and longitudinal studies will be required to determine whether cardiac readouts in EAMG represent direct tissue involvement or adaptive physiological changes (26–28). The reduction in sagittal spinal angle provides an additional layer of phenotypic information. Postural changes in diseased mice are often recognized qualitatively, but objective quantification is less frequently incorporated into EAMG studies. In our study, micro-CT–based sagittal angle measurement provided an imaging-derived structural correlate of the abnormal posture seen clinically. This finding is consistent with the concept that sustained muscle weakness can alter axial loading and postural stability, and it may also relate to previously reported skeletal consequences of EAMG, including reduced bone mineral density (29). Although we deliberately interpreted this measure as a posture-related imaging phenotype rather than structural deformity *per se*, its consistent alteration in the phenotype-enriched subset supports the value of posture-related imaging readouts as exploratory phenotypic measures. The most significant limitation of this study arises from the phenotype-enriched design of the imaging subset. Because echocardiography, PET/CT, and micro-CT were not performed in all animals, and because EAMG mice were preferentially selected on the basis of overt disease manifestations, the imaging subset was non-random and subject to selection bias. This design may inflate the apparent magnitude and frequency of imaging-derived differences compared with an unselected EAMG cohort. Consequently, the imaging findings should be interpreted strictly as exploratory characterizations of a phenotype-enriched subset, not as representative features of the EAMG model as a whole. Additional limitations should also be acknowledged. No formal *a priori* power calculation was performed. The full-cohort sample size was based on prior experimental practice in the EAMG literature, feasibility of multi-domain phenotyping, and animal welfare considerations. The imaging subset was constrained by imaging cost, scanner availability, and animal tolerance for multimodal assessment, and was not designed or powered for definitive group-level inference. Therefore, effect estimates, correlations, and nominal *P* values from this subset should be interpreted cautiously. Multiple exploratory correlation analyses were performed without formal correction for multiplicity; exact *P* values were reported, but nominally significant findings should not be considered confirmatory. Only female C57BL/6J mice were used, and the results may not fully generalize to other strains, sexes, or EAMG induction protocols. Finally, this study was designed as a phenotyping investigation and did not include interventional or mechanistic validation experiments. Therefore, the findings should not be interpreted as demonstrating causal pathways linking immune activation, neuromuscular dysfunction, and imaging-derived organ-level phenotypes. Despite these limitations, the present study supports a more integrated approach to EAMG assessment. Rather than relying on single endpoints, combining electrophysiological, behavioral, immunological, and selected imaging-derived measures may provide a useful framework for tracking disease progression and evaluating treatment effects in preclinical MG studies.

## 5 Conclusion

In conclusion, the classical EAMG model consistently manifested coordinated electrophysiological, functional, and immunological abnormalities in the full cohort. Electrophysiological impairment showed the strongest association with disease severity. Exploratory imaging in a phenotype-enriched subset further identified selected cardiac, metabolic, and postural phenotypes in animals with overt disease manifestations, although these associative findings require validation in systematically imaged, unselected cohorts. Together, these findings support a multi-dimensional phenotyping framework for comprehensive disease profiling in preclinical myasthenia gravis research.

## Data Availability

The original contributions presented in the study are included in the article/Supplementary material, further inquiries can be directed to the corresponding author.

## Supplementary material

Supplementary Figure S1 Representative phenotypic features of EAMG mice. **(A)** Postural abnormalities observed in EAMG mice. **(B)** Representative electromyographic decrement traces. **(C)** Sagittal spinal alignment assessed by micro-CT. **(D)** Reduced skeletal muscle FDG uptake on PET/CT. **(E)** Representative echocardiographic images. These images illustrate typical features observed in EAMG mice and are not intended for quantitative comparison.

Supplementary Figure S2 Additional analyses of disease severity and phenotypic readouts **(A)** Correlation between Lennon score and hanging endurance. **(B)** Correlation between Lennon score and Th17/Treg ratio. **(C)** Correlation between Lennon score and serum anti-AChR levels. Each dot represents one animal. Correlation coefficients were calculated using Spearman's rank correlation.

Supplementary Figure S3 Associations between imaging-derived parameters and disease severity **(A)** Ejection fraction versus Lennon score. **(B)** Heart rate versus Lennon score. **(C)** Iliopsoas FDG uptake versus Lennon score. **(D)** Sagittal spinal angle versus Lennon score. Each dot represents one animal. Spearman correlation was used. Only iliopsoas FDG uptake showed a statistically significant association with disease severity; other parameters did not reach significance.

Supplementary Table S1 Core phenotypes in CFA and EAMG mice at day 90.

Supplementary Table S2 Correlations between disease severity and phenotypic readouts in EAMG mice.

Supplementary Table S3 Imaging-derived phenotypes in the phenotype-enriched subset.
