# Supplementary figures and images for: A multi-dimensional phenotyping framework reveals coordinated neuromuscular, immune, and imaging-derived phenotypes in experimental autoimmune myasthenia gravis
Source: Front Neurol. 2026 Jul 20;17:1857888. doi: 10.3389/fneur.2026.1857888 (PMC13429450; doi:10.3389/fneur.2026.1857888)

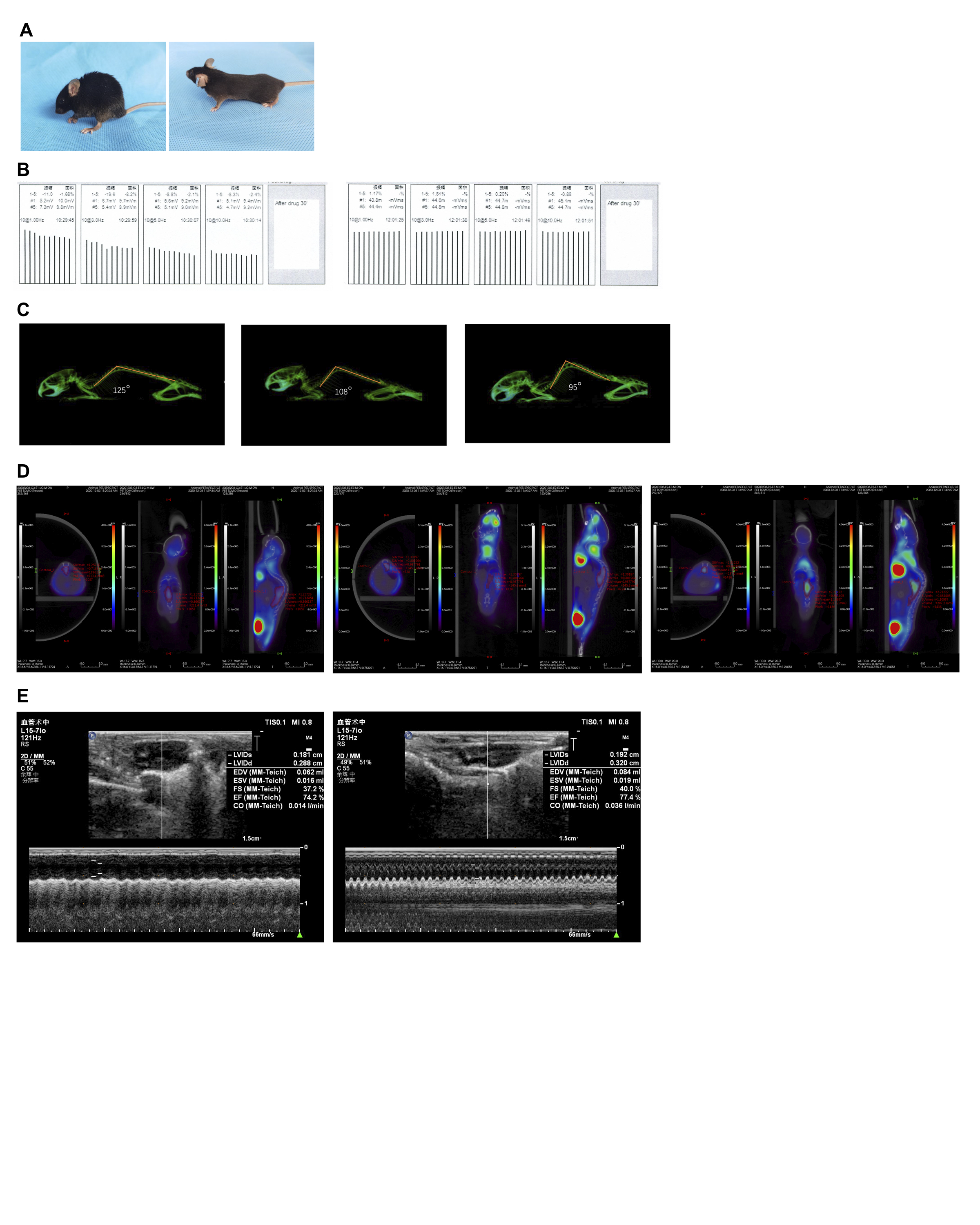

Supplement: Supplementary Figure S1 — Representative phenotypic features of EAMG mice. (A) Postural abnormalities observed in EAMG mice. (B) Representative electromyographic decrement traces. (C) Sagittal spinal alignment assessed by micro-CT. (D) Reduced skeletal muscle FDG uptake on PET/CT. (E) Representative echocardiographic images. These images illustrate typical features observed in EAMG mice and are not intended for quantitative comparison. [file Image_1.TIFF]

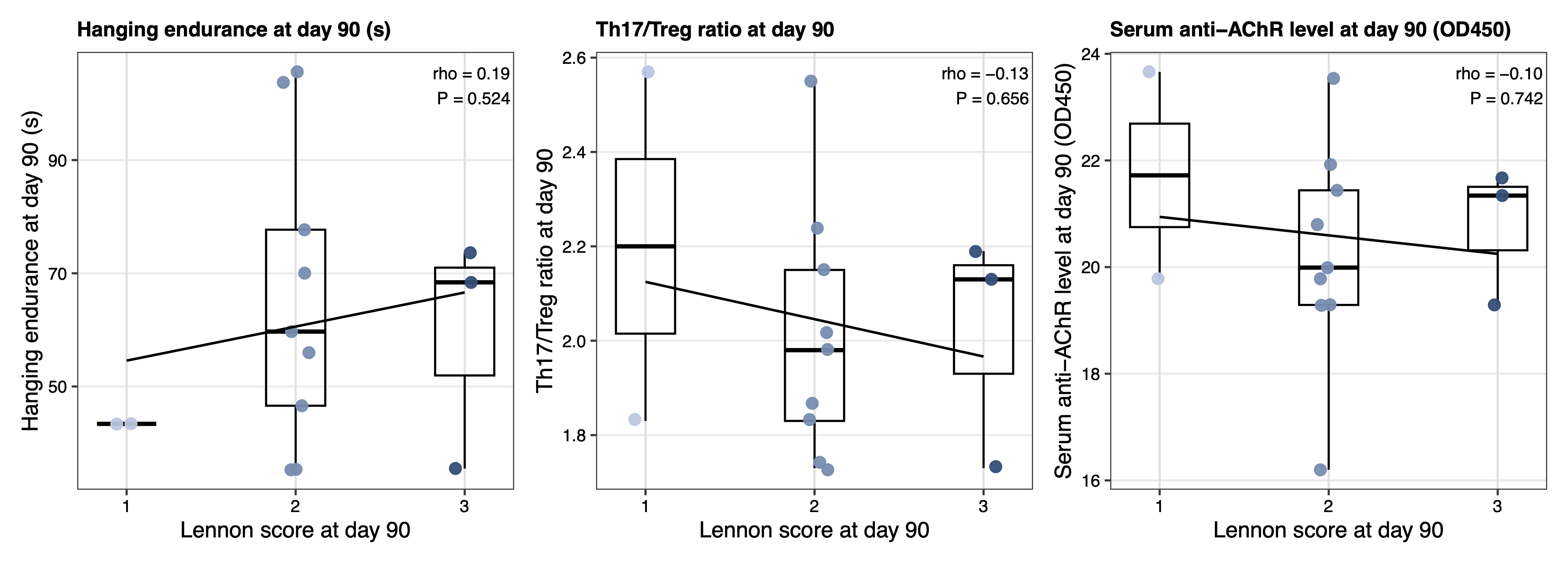

Supplement: Supplementary Figure S2 — Additional analyses of disease severity and phenotypic readouts (A) Correlation between Lennon score and hanging endurance. (B) Correlation between Lennon score and Th17/Treg ratio. (C) Correlation between Lennon score and serum anti-AChR levels. Each dot represents one animal. Correlation coefficients were calculated using Spearman's rank correlation. [file Image_2.TIFF]

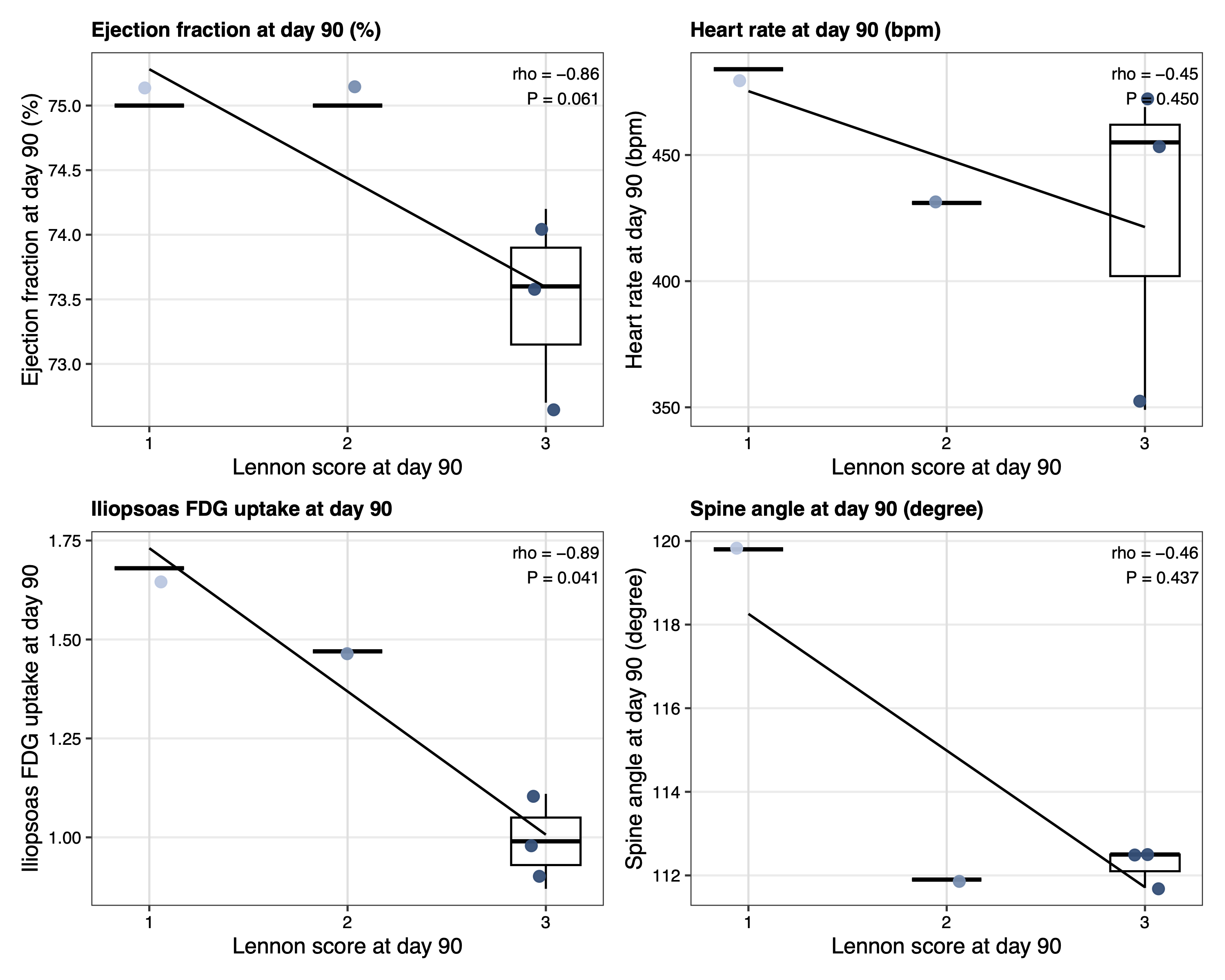

Supplement: Supplementary Figure S3 — Associations between imaging-derived parameters and disease severity (A) Ejection fraction versus Lennon score. (B) Heart rate versus Lennon score. (C) Iliopsoas FDG uptake versus Lennon score. (D) Sagittal spinal angle versus Lennon score. Each dot represents one animal. Spearman correlation was used. Only iliopsoas FDG uptake showed a statistically significant association with disease severity; other parameters did not reach significance. [file Image_3.TIFF]
